# Ebstein anomaly in children—is the cone procedure a valid option?

**DOI:** 10.1093/icvts/ivad044

**Published:** 2023-03-15

**Authors:** Julie Cleuziou

**Affiliations:** Department of Congenital and Paediatric Heart Surgery, German Heart Center Munich, Technical University of Munich, School of Medicine, Munich, Germany

**Keywords:** Ebstein anomaly, Paediatric, Surgery

The Ebstein anomaly (EA) is so rare that it is difficult to find valid publications, analysing all unanswered questions of it [1]. Furthermore, this anomaly of the tricuspid valve is so heterogeneous, that it remains challenging to draw adequate consequences from results. A neonate with a duct-dependent pulmonary perfusion cannot be compared to a 50-year-old without symptoms, although they have the same diagnosis and maybe similar echocardiographic findings. Time has come, in which we have to consider patients with EA depending on their age.

In their report of 188 children below 18 years of age [2], Liu *et al.* review the largest cohort of children with EA undergoing tricuspid valve repair. In other parts of the world, it is hard to reach these numbers of operations, because there are not these numbers of patients. On the other hand, in Asia, other limitations are relevant such as restrictive health services, which influences results, if patients cannot afford surgery even if it might be indicated as reported here. A majority of the patients underwent a cone repair (CR) and the authors demonstrated better outcomes regarding reoperation rate and recurrent tricuspid regurgitation with CR than with other valve repair techniques [2].

Since the publication by da Silva *et al.* [3], we have found a good technique for reconstructing the tricuspid valve in EA with reproducible mid-term results [4, 5]. What is missing are age-dependent results and the answer to the most important question on what is the right time to repair the tricuspid valve. However, there is a trend towards repairing the valve at an earlier stage [6] but still, large cohorts with an emphasis on age are seldom but required to draw adequate consequences.

The authors of this article did not aim at finding the right timing for the CR in paediatric EA. However, their results demonstrate that the CR is the best option in a paediatric population. The remaining question on the right timing for CR is difficult to resolve and will also be dependent on the symptoms of the patients. On the other hand, patients with severe symptoms will probably get earlier surgery. Age might not help in this regard, and the focus should be set on the underlying anatomy. Still, patients with a similar anatomy don’t always present with the same symptoms. Furthermore, what does it mean to be symptomatic in EA? To have a cyanosis? To be less tolerant to exercise? How can you measure exercise tolerance in a 2-year-old child? One could rely on objective data from echocardiography and especially the progress of right ventricular function and tricuspid valve function, like the authors of this study did. But do we know, what the criteria are? How bad must be the right ventricle (RV), to have an indication for surgery? Unfortunately, the weakest point of this study are the missing MRI data, which might have been more precise than echocardiographic data and help in defining criteria for surgery. Conversely, we now have new arguments for an early tricuspid valve repair, due to the encouraging results of the CR.

Another unresolved issue is the need for an additional bidirectional cavopulmonary connection (BCPC), and this might be actually dependent on age. In the current study, the rate of BCPC was 28%, which is similar to the 30% of the paediatric cohort of the Mayo Clinic [4] and much higher than the 6% of the Boston group [6]. The difference might lie within the age of the patients’ population. The authors described some criteria when they performed a BCPC, relying on the size of the atrialized RV, the degree of abnormality of the septal leaflet and the intraoperative haemodynamic instability. The most important risk factor for a BCPC was the degree of displacement of the tricuspid valve. However, the only reproducible reason for a BCPC seems to be the haemodynamic instability. In this circumstance, it will unload the RV, improve its function and probably the outcome. Conversely, we could ask the question: why not always performing a BCPC? Maybe it could save a lot of patients without any big disadvantages [7].

Another debate is the need to plicate the atrialized RV, to reduce its size. Liu *et al.* performed it in about 60% of the patients, other authors have similar rates with 55% [6] and 65% [4]. Mostly, authors do not explain the reason of plicating the atrialized RV or not. The degree of thinning of the myocardium could be important in performing the plication, on the other hand, the proximity of the right coronary artery could preclude a plication. The appreciation of the plication will have to be examined with cardiac MRI, where the size and function of the RV can be very well analysed.

Another subject which needs to be discussed is whether to leave an atrial shunt or not. da Silva *et al.* [3] always leave a small ostium or even creates one if not present, to overcome the early postoperative phase which can be marked by impaired RV function. On the other hand, an atrial shunt can be a risk for emboli in the future and might need an interventional closure. In the present study, a complete closure of an intra-atrial shunt was performed in only 13%, whereas the Mayo Clinic closed the shunt in 67% of the paediatric population [4].

Overall, we are still at the beginning of understanding EA and what is the best treatment, when to perform it and how to perform it. This study has underlined that the CR is the best surgical procedure for all patients with EA, excluding the neonates. Details of timing, additional unloading of the RV, plication of the RV and closing the intra-atrial shunt will need to be answered in the next decades, alongside with long-term results of the CR in different age groups. We need to find out what the real criteria are to improve the life quality and the life expectancy of these patients.
